# Comparing the efficacy of cipaglucosidase alfa plus miglustat with alglucosidase alfa for late-onset Pompe disease: an expanded network meta-analysis utilizing patient-level and aggregate data

**DOI:** 10.57264/cer-2025-0174

**Published:** 2026-02-27

**Authors:** Shuai Fu, Noemi Hummel, Simon Shohet, Neil Johnson, Alasdair MacCulloch, Jeff Castelli, William Kerr, Brian Fox, Vera Gielen

**Affiliations:** 1 Huaiyin Institute of Technology, Huai'an, Jiangsu, China; 2 Certara GmbH, Lörrach, Germany; 3 Amicus Therapeutics Ltd, Marlow, UK; 4 Amicus Therapeutics, Inc., Princeton, NJ, USA

**Keywords:** enzyme replacement therapy, indirect treatment comparison, lysosomal disorder, meta-analysis, metabolic myopathy, multilevel network meta-regression, Pompe disease

## Abstract

**Aim:**

Treatment options for late-onset Pompe disease (LOPD) include enzyme replacement therapy (ERT) with alglucosidase alfa (alg), cipaglucosidase alfa plus miglustat (cipa + mig) and avalglucosidase alfa. However, only one randomized controlled trial (RCT) directly compared cipa + mig and alg and had relatively few ERT-naive patients. A multilevel network meta-regression (ML-NMR) integrated individual patient data and aggregate data into indirect treatment comparisons, with relative effects adjusted to any target population, to compare the efficacy of cipa + mig and alg.

**Materials & methods:**

A Bayesian ML-NMR was conducted to compare the efficacy of cipa + mig and alg for 6-minute walk distance (6MWD, meters) and percent predicted forced vital capacity (ppFVC) across any target population, using patient-level and aggregate data from RCTs (PROPEL, COMET, LOTS) and phase I/II and open-label extension (OLE) trials (PROPEL OLE, LOTS OLE, COMET OLE, ATB200-02, NEO-1/NEO-EXT), adjusting for baseline covariates. Relative effect estimates were obtained for 6MWD and ppFVC change from baseline to week 52. Two networks were analyzed: network A (RCTs only) and network B (RCTs and single-arm OLE and phase I/II studies matched to comparator arms). To assess the impact of prior ERT exposure, simulations were conducted by only varying ERT duration among included covariates.

**Results:**

For cipa + mig compared with alg, both networks were associated with relative increases in 6MWD (mean difference [95% credible interval], Bayesian probability for network A: 13.48 m [6.79, 19.85], >99.9%; network B: 12.59 m [7.89, 17.45], >99.9%) and ppFVC (network A: 1.63% [0.71, 2.60], >99.9%; network B: 3.17% [2.53, 3.81], >99.9%). Network B suggested cipa + mig was favorable (>99.9%) in all groups for both end points and appeared more favorable with increasing ERT duration.

**Conclusion:**

Cipa + mig was associated with an improvement in 6MWD and ppFVC relative to alg independent of prior ERT exposure, which appeared more favorable when all available evidence was used. These data could inform decision-making in treating ERT-naive and ERT-experienced patients with LOPD.

## Plain language summary

**What is this article about?:**

People with late-onset Pompe disease (LOPD) can lose muscle strength and develop breathing difficulties over time. The first drug used to treat LOPD was alglucosidase alfa (alg); however, people often see worsening health after a few years of treatment with alg. Two newer treatment options have recently been approved: avalglucosidase alfa (aval) and cipaglucosidase alfa plus miglustat (cipa + mig).

This study uses a statistical method to combine data from several different clinical studies into a network to compare how well cipa + mig works compared with alg. This study uses the same method as a previous study comparing cipa + mig with aval.

**What were the results?:**

Outcomes included the distance someone can walk in 6 minutes (6-minute walk distance) and how much air someone can forcefully expel from their lungs (forced vital capacity). Results indicated that, when considering all available evidence, treatment with cipa + mig was associated with an improvement compared with alg, whether the participants had previously been treated with alg or not.

**What do the results mean?:**

Although one clinical trial has tested cipa + mig and alg in adults with LOPD, there was a low number of participants who had not had any treatment before starting that trial. Therefore, a network including all available data supports and strengthens the conclusion that cipa + mig is a more favorable treatment than alg in terms of 6-minute walk distance and forced vital capacity. This favorability is seen in people who have not been treated before and those treated with alg previously.

## Pompe disease

Pompe disease is a progressive disease that can lead to irreversible muscle damage due to deficiency of the enzyme acid α-glucosidase (GAA), which hydrolyzes lysosomal glycogen [1]. Due to an accumulation of glycogen in skeletal muscle, patients with Pompe disease are subject to progressive loss of muscular and respiratory function [2]. Pompe disease is typically divided into two categories: infantile-onset Pompe disease, which presents in the first months after birth and progresses rapidly, and late-onset Pompe disease (LOPD), which is progressive and exhibits at variable ages [1,3].

Pharmacological treatment for slowing the progression of LOPD involves enzyme replacement therapy (ERT) with recombinant human GAA (rhGAA). Alglucosidase alfa (alg) was the first of these treatments to be approved [4,5] and was, until recently, the standard of care, though many patients treated with alg experience a decline in outcomes over time [6,7]. Recent advances have seen the approval of two next-generation ERTs – cipaglucosidase alfa in combination with the oral enzyme stabilizer miglustat (cipa + mig) and avalglucosidase alfa (aval) [2,8–10].

## Indirect treatment comparison (ITC)

Due to a lack of direct treatment comparisons for the next-generation ERTs, an ITC has previously been used to compare the treatments cipa + mig and aval [11]. A multilevel network meta-regression (ML-NMR) was conducted to utilize available patient-level and aggregate data from existing clinical trials to determine the relative effect of treatments indirectly while adjusting for multiple baseline covariates, including prior ERT exposure, to account for differences in patient populations across studies [11,12]. As this focused on cipa + mig and aval, trials not containing these interventions were excluded from the analysis. Here, additional clinical trial evidence in LOPD containing only alg or alg and placebo has been included for a more robust comparison of cipa + mig and alg in an ML-NMR analysis.

Only one randomized controlled trial (RCT) has been conducted to date that directly compares cipa + mig and alg (PROPEL) [2], with a small number of treatment-naive patients (n = 28; 23% of the total PROPEL population), 20 (16% of the total PROPEL population) randomized to cipa + mig and eight (7% of the total PROPEL population) to alg + placebo. An ITC that analyzes all available evidence for these treatments could help address this data gap.

## Objectives

This study used previously outlined ML-NMR methodology, updated to include all publicly available clinical trial data for cipa + mig and alg, to compare the efficacy of cipa + mig and alg in terms of 6-minute walk distance (6MWD, meters) and percent predicted forced vital capacity (ppFVC). This simulation scenario included both base case and prior ERT experience scenarios. In the base case scenario, all covariates considered to be potential treatment effect modifiers were set to the target population of the PROPEL trial.

## Materials & methods

The network meta-analysis followed the methodology of Shohet *et al.* [11], which compared cipa + mig with aval. Note that, while aval was included in the network analyses, it is not the focus of this manuscript.

### Systematic literature review

In the previous publication, a systematic literature review was conducted to identify relevant studies on the efficacy of cipa + mig and alg [11]. The studies included in Shohet *et al.* [11] were also used in the present analysis, as well as two previously excluded trials, LOTS and LOTS open-label extension (OLE), which only have alg as an intervention. Data on trial details, patient characteristics, treatments and outcomes were extracted from the identified studies and critically appraised using the revised tool for Risk Of Bias in Randomized Studies and the Risk Of Bias In Non-randomized Studies tool for interventional non-RCTs [13,14]. Relevant efficacy results over time were extracted and used in the ML-NMR analysis with both networks. This analysis focused on the relative effect at week 52; however, many of the included studies had extensions beyond 52 weeks of treatment, and data for 6MWD and ppFVC at other time points (past 52 weeks) were included in this analysis and are summarized in Supplementary Figure 1.

### ML-NMR

Using patient-level and aggregate published data from RCTs (PROPEL [NCT03729362] [2], LOTS [NCT00158600] [5] and COMET [NCT02782741] [8]) and open-label extension (OLE) and phase I/II trials (PROPEL OLE [NCT04138277] [15], LOTS OLE [NCT00455195] [16], COMET OLE [NCT02782741] [17], ATB200-02 [NCT02675465] [18] and NEO-1/NEO-EXT [NCT01898364/NCT02032524] [19]), a Bayesian ML-NMR was conducted to obtain relative effect estimates on 6MWD and ppFVC [12,20]. Due to the timing of these studies, it was only possible for patients to switch from alg to cipa + mig or aval, thus impacting the structure of the network and meaning that it was not possible to compare all possible treatment sequences with the available data.

The ML-NMR adjusted for various baseline covariates to address heterogeneity of the trial populations, including age, sex, baseline outcome values and prior ERT duration [7], the latter being an important treatment effect modifier. Covariates were selected as described in Shohet *et al.* [11]. While there is potential for interactions between covariates, these were not investigated in this analysis. Two networks were analyzed: firstly, network A, which included RCT evidence only. In a second network (network B), all published single-arm OLE and phase I/II studies were included in addition to the RCT evidence included in network A. For network B (all evidence), single-arm study results were matched to the appropriate comparator arms of the RCTs for inclusion into the network, based on minimizing the differences between the two arms. Matching was performed as described in Shohet *et al.* [11]. COMET OLE and NEO-1/NEO-EXT were matched to alg from COMET; ATB200-02 and PROPEL OLE were matched to alg from PROPEL. In addition to the previous ML-NMR, LOTS OLE was matched to placebo from LOTS. Mean treatment differences were calculated for 6MWD (meters) and ppFVC change from baseline at week 52 [21,22].

The Bayesian probability that the treatment was superior (mean difference >0) or inferior (mean difference <0) to the reference treatment was derived as previously described [11]. No strict threshold for interpreting these Bayesian probabilities was adopted in line with guidance from the American Statistical Association, and the probability itself was reported [23,24]. Probabilities were interpreted as suggesting strong evidence (probability <0.1% or >99.9%), evidence (probability between 0.1% and 5% or between 95% and 99.9%), weak evidence (probability between 5% and 10% or between 90% and 95%) and no evidence.

A base case scenario was evaluated in which covariates were set to the target population of the PROPEL trial (which included a mixed population of ERT-naive and ERT-experienced patients with LOPD reflective of the real-world population) [2]: mean age = 46.95 years, male = 45.08%, mean ERT duration = 5.744 years, mean 6MWD = 355.8 m, mean ppFVC = 70.42%, time = 52 weeks.

To study the impact of ERT duration on relative effects, the ERT duration was varied, keeping the remaining covariate values as in the base case scenario. Prior ERT durations were determined based on the PROPEL study [2], where 0 years was the minimum prior ERT duration (ERT naive), and 2.5 years (short) and 9.2 years (long) represented the first and third quartiles of baseline ERT duration, respectively. A scenario of 5 years (medium) was included to explore the point at which the relative effect of cipa + mig versus alg becomes favorable, noting that the median prior ERT duration in the individual PROPEL patient data was 6.1 years (for all patients, including ERT-naive patients as 0 years).

Fixed effects ML-NMR models were applied, as determined appropriate by Shohet *et al.* due to the limited number of studies included in the network that could impact the between-study variability [25].

## Results

### Systematic literature review

**Figure 1.**
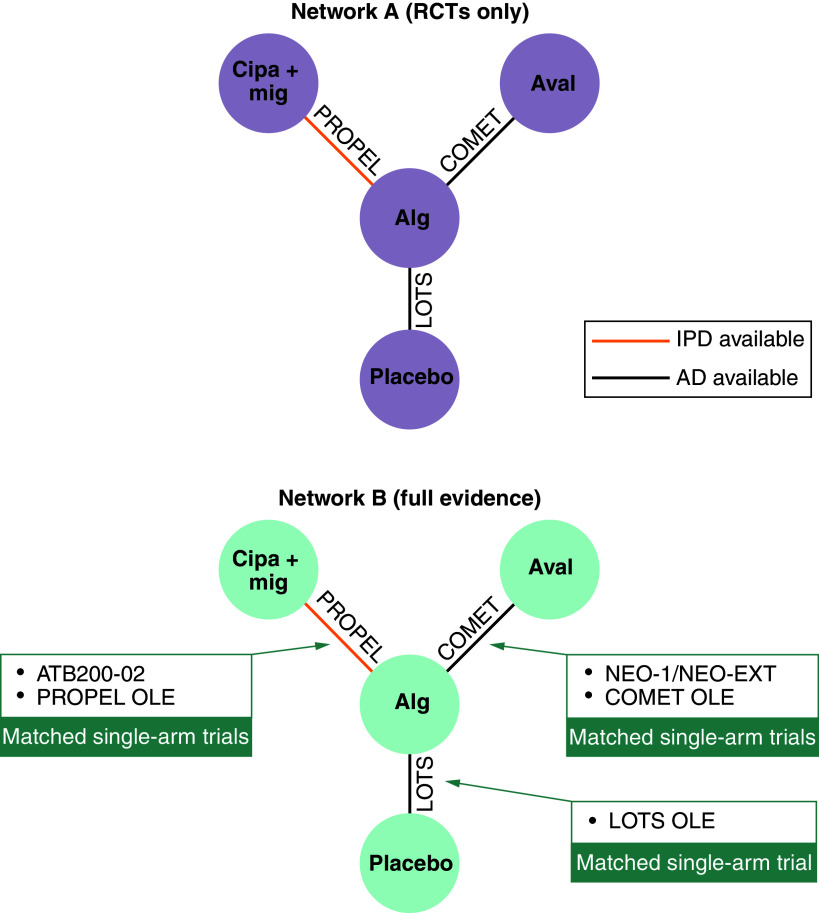
Networks for 6-minute walk distance (meters) and percent predicted forced vital capacity. Figure adapted from Shohet *et al.* 2024. AD: Aggregate data; alg: Alglucosidase alfa; aval: Avalglucosidase alfa; cipa + mig: Cipaglucosidase alfa plus miglustat; EXT: Extension; IPD: Individual patient-level data; OLE: Open-label extension; RCT: Randomized controlled trial.

As seen in Figure 1, network A contained RCTs only (PROPEL [2], LOTS [5], COMET [8]), while additional single-arm trials were subsequently matched to the RCTs in network B (all evidence) for the full evidence analysis (LOTS OLE [16], ATB200-02 [18], PROPEL OLE [15], NEO-1/NEO-EXT [19], COMET OLE [17]). In addition to the studies previously used in Shohet *et al.* the LOTS study, which compared alg with placebo, and the LOTS single-arm OLE were included [5,16]. Trial and baseline characteristics for the included studies are shown in Table 1. Longitudinal efficacy results were extracted from each study for the shared end points of mean change in 6MWD and ppFVC from baseline (Supplementary Figure 1).

**Table 1.** Trial and patient baseline characteristics of included trials.

Trial	Treatment	Population	n	Age (years), mean (SD)	Male (%)	ERT (years), mean (SD)	6MWD (meters), mean (SD)	ppFVC, mean (SD)
NEO-1/NEO-EXT	Aval	ERT naive	10	44.8 (20.3)	30.0	0.0 (0.0)	449.2 (118.4)	69.2 (19.3)
		ERT experienced	14	46.7 (14.1)	64.3	4.0 (2.0)	440.4 (141.0)	77.3 (16.5)
COMET	Alg	ERT naive	49	50.3 (13.7)	51.0	0.0 (0.0)	378.1 (116.2)	61.6 (12.4)
	Aval	ERT naive	51	46.0 (14.5)	52.9	0.0 (0.0)	399.3 (110.9)	62.5 (14.4)
COMET OLE	Aval	ERT experienced	44	51.2 (13.7)^†^	51.0^‡^	0.9 (0.0)	383.6 (141.1)^†^	61.2 (13.5)^†^
ATB200-02	Cipa + mig	ERT experienced	17	45.7 (36.0)	64.7	6.4 (1.3)	393.7 (119.6)	57.1 (17.9)
		ERT naive	6	46.1 (39.1)	16.7	0.0 (0.0)	396.0 (75.2)	55.8 (19.1)
PROPEL	Cipa + mig	ERT experienced	65	47.6 (13.3)	42.4	7.5 (3.4)	346.9 (110.2)	67.9 (19.1)
		ERT naive	20			0.0 (0.0)	393.6 (112.4)	80.2 (18.7)
	Alg	ERT experienced	30	45.4 (13.4)	51.4	7.1 (3.6)	334.6 (114.0)	67.5 (21.0)
		ERT naive	8			0.0 (0.0)	420.9 (135.7)	79.1 (22.6)
PROPEL OLE	Cipa + mig	ERT experienced	37	48.6 (13.3)	51.4	6.7 (4.3)	359.7 (137.4)	62.8 (20.5)
LOTS	Alg	ERT naive	60	45.3 (12.4)	57.0	0.0 (0.0)	332.2 (126.7)	55.4 (14.4)
	Placebo	ERT naive	30	42.6 (11.6)	37.0	0.0 (0.0)	317.9 (132.3)	53.0 (15.7)
LOTS OLE	Alg	ERT naive	26	44.1 (11.6)^‡^	37.0^†^	0.0 (0.0)	312.7 (147.2)^†^	51.1 (15.8)^†^

† Re-baselined after the double-blind period.

‡ Same percent male assumed as in the double-blind period.

6MWD: 6-minute walk distance; alg: Alglucosidase alfa; aval: Avalglucosidase alfa; cipa + mig: Cipaglucosidase alfa plus miglustat; ERT: Enzyme replacement therapy; OLE: Open-label extension; ppFVC: Percent predicted forced vital capacity; SD: Standard deviation.

### Relative effects estimation based on ML-NMR

#### Base case scenario

**Figure 2.**
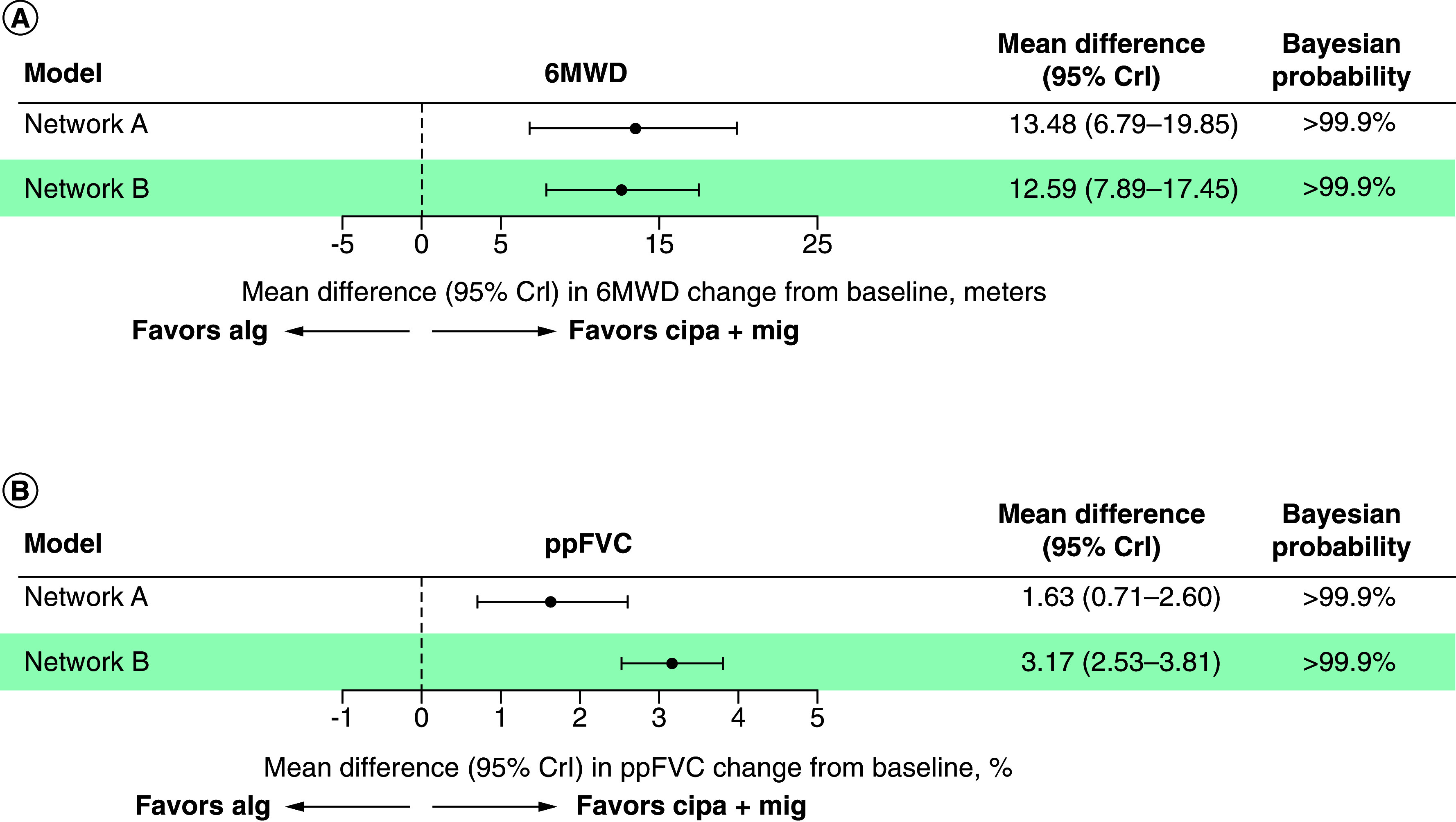
Base case forest plots. **(A)** 6MWD (meters). **(B)** ppFVC. 6MWD: 6-minute walk distance; alg: Alglucosidase alfa; aval: Avalglucosidase alfa; cipa + mig: Cipaglucosidase alfa plus miglustat; CrI: Credible interval; ppFVC: Percent predicted forced vital capacity.

In the base case scenario (baseline covariates as in PROPEL [2]), which included all covariates, analysis of network A (RCTs only) suggested that cipa + mig, when compared with alg, was more favorable, with strong evidence supporting an association with improvement in 6MWD (mean difference 13.48 m [95% credible interval (CrI): 6.79, 19.85], Bayesian probability >99.9%) and ppFVC (mean difference 1.63% [95% CrI: 0.71, 2.60], >99.9%; Figure 2). Analysis of network B (all evidence) was also associated with favorability, providing strong evidence for cipa + mig in 6MWD (mean difference 12.59 m [95% CrI: 7.89, 17.45], >99.9%) and ppFVC (mean difference 3.17 % [95% CrI: 2.53, 3.81], >99.9%).

#### Impact of prior ERT exposure on relative effects

**Figure 3.**
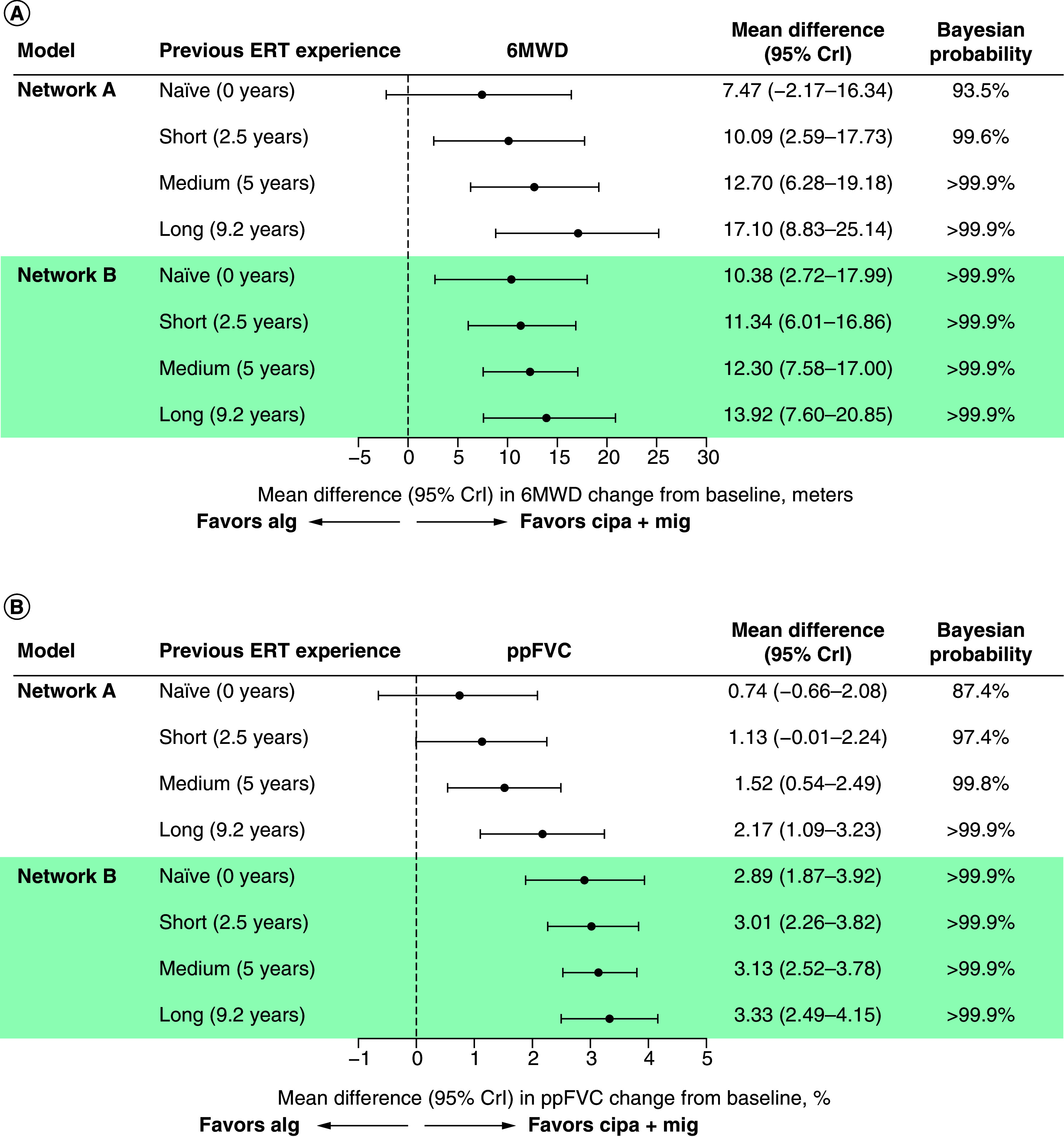
Forest plots by prior ERT exposure. **(A)** 6MWD (meters). **(B)** ppFVC. 6MWD: 6-minute walk distance; alg: Alglucosidase alfa; aval: Avalglucosidase alfa; cipa + mig: Cipaglucosidase alfa plus miglustat; CrI: Credible interval; ERT: Enzyme replacement therapy; ppFVC: Percent predicted forced vital capacity.

Figure 3 shows the effect of prior ERT exposure when comparing cipa + mig with alg. In network A (RCTs only), cipa + mig was favorable with strong evidence for 6MWD in scenarios of long and medium prior ERT duration (Bayesian probability >99.9%), and evidence in the short prior ERT duration scenario (99.6%). For ppFVC, cipa + mig was favorable with strong evidence for long prior ERT duration (>99.9%) and evidence for medium and short prior ERT duration scenarios (99.8% and 97.4%, respectively). In the scenario where prior ERT duration was 0 years, cipa + mig was favorable, but evidence for improvement in 6MWD (93.5%) and ppFVC (87.4%) was not considered strong.

For network B (all evidence), all prior ERT duration scenarios, including ERT naive, were favorable with strong evidence (>99.9%) for cipa + mig versus alg in 6MWD and ppFVC. Mean difference increased with prior ERT duration for both 6MWD and ppFVC, suggesting greater favorability for cipa + mig with increasing prior ERT duration.

## Discussion

In the base case scenario, cipa + mig was more favorable than alg in 6MWD and ppFVC change from baseline measures when considering RCT only (network A) and all evidence (network B). Considering prior ERT exposure, when all evidence was included in the analysis (network B), cipa + mig appeared to be associated with greater improvements in all instances when compared with alg, with strong evidence in both 6MWD and ppFVC regardless of prior ERT exposure. When only RCTs were included (network A), cipa + mig appeared to demonstrate a favorable mean treatment difference versus alg across all groups. This was most pronounced in patients with longer prior ERT exposure. In the ERT-naive scenario, while the Bayesian probability determined no evidence for the favorability of cipa + mig versus alg in network A, strong evidence for the favorability of cipa + mig over alg was demonstrated in the ERT-naive scenario in network B when all evidence was included.

The strong evidence observed in network B for both 6MWD and ppFVC, regardless of prior ERT exposure, agrees with Shohet *et al.* where cipa + mig had the highest probability of being ranked as best among treatments when all evidence was considered [11]. In the PROPEL study, cipa + mig was compared directly with alg in a mixed population of adult patients who were either ERT experienced or ERT naive, reflecting real-world conditions. A pre-specified analysis of the larger cohort of ERT-experienced patients (77% of the study population) indicated that participants randomized to cipa + mig (n = 65) experienced clinically meaningful improvements from baseline to week 52 in 6MWD and ppFVC compared with those randomized to alg + placebo (n = 30) [2]. The relative effects of the present study, using the full analysis network, are aligned with these findings. For ERT-naive patients in PROPEL (23% of the study population), a pre-specified analysis demonstrated that there were no differences in 6MWD and ppFVC (change from baseline to week 52) between those randomized to cipa + mig (n = 20) and those randomized to alg + placebo (n = 8) [2]. In the present study, with the inclusion of additional available evidence for cipa + mig and alg, improvements and favorability for cipa + mig versus alg were observed for all groups for both 6MWD and ppFVC in network B (all evidence). This may be due to the inclusion of evidence from additional studies, including ATB200-02, COMET, LOTS and LOTS OLE, which introduced a larger population of ERT-naive patients treated with alg [5,16]. Given the relatively small population of ERT-naive patients in head-to-head trials to date, incorporating all available evidence allows for a more comprehensive analysis and helps to better reflect the diversity of real-world populations.

The relative differences between cipa + mig and alg observed here are consistent with those observed in the head-to-head PROPEL RCT, which showed a between-group 6MWD difference of 13.6 m in favor of cipa + mig over alg at week 52 [2], comparable to mean differences of 13.48 m and 12.59 m observed here in the base case scenario for networks A and B, respectively. Similarly, the PROPEL trial reported a mean difference in ppFVC of 3.0% in cipa + mig versus alg [2]; here, we observed similar mean differences in ppFVC of 1.63% (network A) and 3.17% (network B).

When assessing the impact of prior ERT exposure on relative effects, it should be considered that all participants in the included trials who switched treatments did so from alg to aval or cipa + mig. This is because, at the time that these studies began, alg was the only available treatment. Thus, no participant switched from cipa + mig to alg, thereby limiting a full comparison of all treatment effects. Nevertheless, this is reflective of current real-world treatment practices in which patients are switching from alg to cipa + mig or aval but not the other way around.

The trend observed here suggests that greater improvements are associated with increasing prior ERT exposure. While this could imply that introducing any new therapy may result in a more pronounced effect, network B analysis of the ERT-naive cohort suggests a favorable effect of cipa + mig over alg even without prior treatment, indicating that the beneficial effect is not solely due to switching therapies. Rather, the observed differences in efficacy seen by different prior ERT duration may be explained by differences in chemistry and mode of action between cipa + mig and alg [26,27]. Cipa is enriched with bis-phosphorylated mannose-6-phosphate, mediating high-affinity binding and effective uptake into muscle cells, ensuring that it can be processed to produce mature rhGAA in the lysosome [27–29]. Cipa is stabilized by mig when in circulation, enhancing the available amount of rhGAA for uptake into cells [26]. This combination may enable cipa + mig to clear accumulated and more highly complexed glycogen in skeletal muscles, particularly benefiting patients who have been undergoing therapy for an extended period. Clinical analysis has shown improvements from baseline to week 52 in motor function, muscle strength, biomarkers and measures of quality of life that were maintained to week 104, and outcomes for pulmonary function were generally stable from baseline to week 104 [15], thereby suggesting that patients may not experience the same diminishing effects with cipa + mig as has been observed with alg.

This analysis shows that cipa + mig, given as the primary treatment to ERT-experienced or ERT-naive patients, could reduce or prevent further decline compared with alg, thereby improving future quality of life for patients.

### Limitations

Limitations to the ML-NMR method were previously discussed in Shohet *et al.* and apply to this analysis. These include that when matching single-arm trials to the comparator arms of the head-to-head RCTs, high heterogeneity between the single and matched arms (outside of matching factors, i.e., baseline age, sex, baseline previous ERT duration, baseline 6MWD or baseline ppFVC) can lead to bias in the relative effect. Another limitation of the ML-NMR method is that it can only adjust the relative effect estimates for any observed effect modifier available in the data, but not for unobservable effect modifiers. While a significant number of potential treatment effect modifiers were included in this analysis, the lack of reporting of modifiers such as genotype in published studies means they could not be included. Despite these limitations, this methodology allows the incorporation of all available evidence on the effectiveness of treatments in heterogeneous patient populations, which would otherwise not be feasible.

## Conclusion

In adults with LOPD, cipa + mig appeared to demonstrate greater improvements in both 6MWD and ppFVC compared with alg, independent of prior ERT exposure, which in most instances became more pronounced (i.e., larger effect sizes) when all available evidence was used in the analysis. This ML-NMR made optimal use of all available evidence, enabling the incorporation of data from trials with patient populations differing in the important treatment effect modifier of prior ERT duration. The inclusion of LOTS into the networks and LOTS OLE into network B (all evidence) further strengthens the ML-NMR analysis, leading to more precise estimates (narrower credible intervals) due to the addition of further evidence on alg. These results could inform decision-making in the treatment of LOPD, as the benefits of cipa + mig compared with alg are evident in both ERT-experienced and ERT-naive patients. Now that these products are available for use, real-world effectiveness can be tested and these findings can be confronted with real-world evidence studies and analyses of registry data, including robust populations of ERT-experienced and ERT-naive patients.

## Summary points

- Only one randomized clinical trial (RCT) directly compares cipaglucosidase alfa plus miglustat (cipa + mig) with alglucosidase alfa (alg), the standard of care for late-onset Pompe disease for over a decade. This trial had a small population of treatment-naive patients, particularly in the alg group.
- An indirect comparison of cipa + mig with alg, utilizing all available data, allows for investigation of favorability by the important treatment modifier of prior enzyme replacement therapy (ERT) experience.
- The multilevel network meta-regression method has been previously used to compare treatments using two networks. Network A, containing only RCTs, and network B, additionally including phase I/II studies and single-arm open-label extension (OLE) studies. The present analysis incorporates further evidence from the LOTS and LOTS OLE studies, contributing evidence on alg treatment effects.
- Analyses included a base case scenario, with covariates set to those of PROPEL, and scenarios varying prior ERT exposure.
- For the base case scenario, both networks indicated that cipa + mig was associated with a favorable relative effect for both 6-minute walk distance (6MWD) and percent predicted forced vital capacity (ppFVC) at week 52 compared with alg.
- When using the full evidence in network B, cipa + mig appeared considerably more favorable than alg, independent of prior ERT experience, with efficacy increasing with longer prior ERT duration.
- The evidence in this analysis indicates that cipa + mig may be more favorable than alg, independent of prior ERT experience, for measures of 6MWD and ppFVC.

## Supplementary Material


