# Supplementary material for: Comparing the efficacy of cipaglucosidase alfa plus miglustat with alglucosidase alfa for late-onset Pompe disease: an expanded network meta-analysis utilizing patient-level and aggregate data
Source: J Comp Eff Res. 2026 Feb 27;15(3):e250174. doi: 10.57264/cer-2025-0174 (PMC12976639; doi:10.57264/cer-2025-0174)
Supplement: Supplementary file 1 [file cer-15-250174-s1.docx]

# **Comparing** the efficacy of cipaglucosidase alfa plus miglustat with alglucosidase alfa for late-onset Pompe disease: an expanded network meta-analysis utilizing patient-level and aggregate data

## Supplementary materials

**
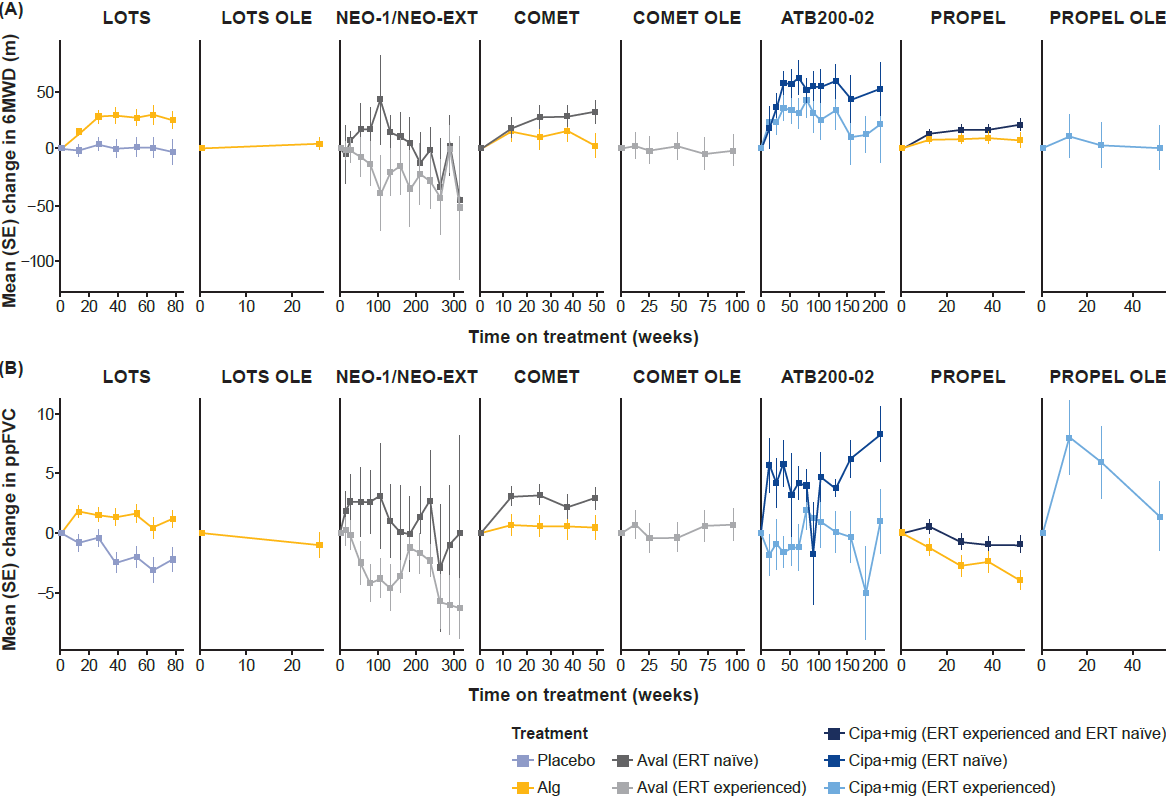
**

**Supplementary Figure 1. Longitudinal efficacy results (mean ± SE) extracted from each study for the shared endpoint of mean change in (A) 6MWD (meters) and (B) ppFVC.**

6MWD: 6-minute walk distance; alg: alglucosidase alfa; aval: avalglucosidase alfa; cipa+mig: cipaglucosidase alfa plus miglustat; ERT: enzyme replacement therapy; EXT: extension; OLE: open-label extension; ppFVC: % predicted forced vital capacity; SE: standard error.
